# Dual therapy with an oral non-vitamin K antagonist and a P2Y12 inhibitor vs triple therapy with aspirin, a P2Y12 inhibitor and a vitamin K antagonist for the treatment of diabetes mellitus patients with co-existing atrial fibrillation following percutaneous coronary intervention: A meta-analysis

**DOI:** 10.1097/MD.0000000000025546

**Published:** 2021-04-16

**Authors:** Qiang Wang, Keping Yang

**Affiliations:** Institute of Cardiovascular Diseases, Jingzhou Central Hospital, Jingzhou Clinical Medical College, Yangtze University, Jingzhou, Hubei, PR China.

**Keywords:** aspirin, atrial fibrillation, bleeding events, diabetes mellitus, dual therapy, major adverse cardiac events, non-vitamin K antagonist, P2Y12 inhibitors, percutaneous coronary intervention, stent thrombosis, triple therapy

## Abstract

**Background:**

In this analysis, we aimed to compare the efficacy and safety of dual therapy (DT) with a non-vitamin K oral anticoagulant (NOAC) and an adenosine diphosphate receptor antagonist (P2Y12 inhibitor) vs triple therapy (TT) with aspirin, a P2Y12 inhibitor and a vitamin K antagonist for the treatment of diabetes mellitus (DM) patients with co-existing atrial fibrillation (AF) following percutaneous coronary intervention (PCI).

**Methods:**

Medical Literature Analysis and Retrieval System Online (MEDLINE), http://www.ClinicalTrials.gov, Excerpta Medical data BASE (EMBASE), Web of Science, Cochrane Central and Google Scholar were the searched databases. Studies that were randomized trials or observational studies comparing DT vs TT for the treatment of DM patients with co-existing AF following PCI were included in this analysis. The adverse cardiovascular outcomes and bleeding events were the endpoints. This meta-analysis was carried out by the RevMan version 5.4 software. Risk ratios (RR) with 95% confidence intervals (CI) were used to represent data and interpret the analysis.

**Results:**

A total number of 4970 participants were included whereby 2456 participants were assigned to the DT group and 2514 participants were assigned to the TT group. The enrollment period varied from year 2006 to year 2018. Our current results showed that major adverse cardiac events (RR: 1.00, 95% CI: 0.84–1.20; *P* = .98), mortality (RR: 1.08, 95% CI: 0.78–1.48; *P* = .66), myocardial infarction (RR: 1.02, 95% CI: 0.74–1.42; *P* = .90), stroke (RR: 0.94, 95% CI: 0.53–1.67; *P* = .84) and stent thrombosis (RR: 1.09, 95% CI: 0.56–2.10; *P* = .80) were similar with DT versus TT in these patients. However, the risks for total major bleeding (RR: 0.66, 95% CI: 0.54–0.82; *P* = .0001), total minor bleeding (RR: 0.74, 95% CI: 0.64–0.85; *P* = .0001), Thrombolysis in Myocardial Infarction (TIMI) defined major bleeding (RR: 0.58, 95% CI: 0.35–0.95; *P* = .03), TIMI defined minor bleeding (RR: 0.62, 95% CI: 0.42–0.92; *P* = .02), intra-cranial bleeding (RR: 0.34, 95% CI: 0.13–0.95; *P* = .04) and major bleeding defined by the International Society on Thrombosis and Hemostasis (RR: 0.68, 95% CI: 0.51–0.90; *P* = .008) were significantly higher with TT.

**Conclusions:**

DT with a NOAC and a P2Y12 inhibitor was associated with significantly less bleeding events without increasing the adverse cardiovascular outcomes when compared to TT with aspirin, a P2Y12 inhibitor and a Vitamin K antagonist for the treatment of DM patients with co-existing AF following PCI. Hence, DT is comparable in efficacy, but safer compared to TT. This interesting hypothesis will have to be confirmed in future studies.

## 1 Introduction

Diabetes mellitus (DM) and cardiovascular disease (CVD) often co-exist.^[1]^ Percutaneous coronary intervention (PCI) has commonly been used to treat patients with CVD.^[2]^ Dual antiplatelet therapy (DAPT) with aspirin and a P2Y12 inhibitor such as clopidogrel or ticagrelor has been used to prevent cardiovascular complications following PCI.^[3]^

However, due to platelet dysfunctions in patients with DM, resulting in platelet hyperactivity,^[4]^ antiplatelets and antithrombotic agents have often been a subject of debate in such patients. Triple therapy with aspirin, an adenosine diphosphate receptor antagonist (P2Y12 inhibitor) and a vitamin K antagonist has also been used to treat patients after PCI.^[5]^

Vitamin K antagonists such as warfarin, which inhibit vitamin K dependent coagulation proteins and thrombin formation have shown to reduce cardiovascular outcomes after a myocardial infarction, but they were apparently associated with excessive bleeding.^[6]^ The combination of vitamin K antagonist with aspirin has also shown to be effective, but with doubtful safety outcomes.^[7]^

Recently, novel antithrombotic agents were introduced. Direct factor Xa inhibitors or non-vitamin K antagonists combined with antiplatelet therapy showed mixed results following PCI.^[8]^ In a study consisting of patients with acute coronary syndrome, apixaban 5 mg twice daily in combination with an antiplatelet agent did not decrease thrombotic events, but however, it increased intracranial and fatal bleedings.^[9]^ In the ATLAS 2 trial, addition of a lower dose of a non-vitamin K antagonist to DAPT reduced major ischemic/thrombotic events but with a moderate risk of bleeding.^[8]^

In this analysis, we aimed to compare the efficacy and safety of dual therapy (DT) with a non-vitamin K oral anticoagulant (NOAC) and a P2Y12 inhibitor versus triple therapy (TT) with aspirin, a P2Y12 inhibitor and a Vitamin K antagonist for the treatment of DM patients with co-existing atrial fibrillation (AF) following PCI.

## 2 Methods

### 2.1 Data sources

Medical Literature Analysis and Retrieval System Online (MEDLINE), http://www.ClinicalTrials.gov, Excerpta Medical data BASE (EMBASE), Web of Science, Cochrane Central and Google Scholar were the searched databases.

### 2.2 Search strategies

The following terms and phrases were searched from the above-mentioned electronic databases:

- Dual therapy vs triple therapy and atrial fibrillation;
- NOAC and atrial fibrillation and percutaneous coronary intervention;
- Oral anticoagulant and atrial fibrillation and coronary stenting;
- Dual vs triple therapy and AF and percutaneous coronary intervention;
- Dual vs triple therapy and AF and diabetes mellitus and percutaneous coronary intervention;
- NOAC and atrial fibrillation and diabetes mellitus and percutaneous coronary intervention;
- Warfarin and percutaneous coronary intervention.

Respective names of the NOAC were also used during the search process:

- Dabigatran and atrial fibrillation and percutaneous coronary intervention;
- Rivaroxaban and atrial fibrillation and percutaneous coronary intervention;
- Apixaban and atrial fibrillation and percutaneous coronary intervention;
- Endoxaban and atrial fibrillation and percutaneous coronary intervention.

### 2.3 Inclusion and exclusion criteria

Criteria for inclusion were:

- Studies that were randomized trials or observational cohorts comparing DT with a NOAC and a P2Y12 inhibitor vs TT with aspirin, a P2Y12 inhibitor and a vitamin K antagonist for the treatment of AF patients following PCI;
- Studies that consisted of patients with DM;
- Studies that reported adverse cardiovascular and bleeding outcomes;
- Studies that were published in English.

### 2.4 Criteria for exclusion were:

Systematic reviews, literature reviews and meta-analyses;

- Case studies;
- Studies that did not compare DT with a NOAC and a P2Y12 inhibitor vs TT with aspirin, a P2Y12 inhibitor and a vitamin K antagonist for the treatment of AF patients following PCI;
- Studies that did not report cardiovascular and bleeding outcomes;
- Studies that were duplicates;
- Studies that consisted of data which could not be used for this analysis.

### 2.5 Definitions and outcomes

Participants in the DT group were treated with a NOAC plus a P2Y12 inhibitor.

Participants in the TT group were treated with aspirin, a P2Y12 inhibitor and a vitamin K antagonist.

Major adverse cardiac events (MACEs)^[10]^ were defined as a combination of all-cause death, myocardial infarction, stroke, and revascularization.

Stent thrombosis was defined according to the Academic Research Consortium (ARC).^[11]^

International Society on Thrombosis and Hemostasis (ISTH) major bleeding^[12]^ has been defined as clinically overt bleeding that is associated with a fall in hemoglobin of 2 g/dl or more, or a transfusion of 2 or more units of packed red blood cells or whole blood or a critical site such as intraocular, intraspinal, intracerebral, pericardial, intra-articular, retroperitoneal, or a fatal outcome.

Thrombolysis in Myocardial Infarction (TIMI) major bleeding^[12]^ has been defined as any symptomatic intracerebral hemorrhage, or clinically overt signs of hemorrhage including imaging, associated with a decrease in hemoglobin of equal to or more than 5 g/dl or an absolute decrease in hematocrit level of ≥15%.

TIMI minor bleeding^[12]^ has been defined as any clinically overt sign of hemorrhage that is associated with a decrease in hemoglobin level of 3 to 5 g/dl or a fall in hematocrit of 9% to 15%.

The adverse cardiovascular outcomes and bleeding events which were reported in the original studies have been listed in Table 1 and the outcomes which have been analyzed included:

- MACEs;
- Myocardial infarction (MI);
- Stroke;
- Mortality;
- Stent thrombosis;
- Total major bleeding;
- Total minor bleeding;
- TIMI defined major bleeding;
- TIMI defined minor bleeding;
- Intracranial bleeding;
- ISTH major bleeding.

**Table 1:** The outcomes and follow-up which were reported in the original studies.

Studies	Outcomes	Types of participants	Follow-up time period (months)	Treatments
Cannon 2017^[16]^	ISTH minor bleeding, total bleeding, intracranial hemorrhage, TIMI major bleeding, TIMI major or minor bleeding, MACEs, death, MI, stroke, definite stent thrombosis	DM + AF + PCI	14 mo	Dabigatran (110 and 150 mg) + P2Y12 inhibitor vs Warfarin + ASA + P2Y12 inhibitor
Gibson 2016^[12]^	Major bleeding, minor bleeding, bleeding requiring medical attention, MACEs, death from cardiovascular causes, MI, stroke, stent thrombosis	General population including DM patients + AF + PCI	12 mo	Rivaroxaban (15 mg and 5 mg) + P2Y12 inhibitor vs Warfarin + ASA + P2Y12 inhibitor
Lopes 2019^[17]^	ISTH major bleeding, death, clinically relevant non-major bleeding, intracranial hemorrhage, GUSTO severe or moderate bleeding, GUSTO severe bleeding, GUSTO moderate bleeding, TIMI major or minor bleeding, TIMI major bleeding, TIMI minor bleeding, death from cardiovascular causes, stroke, MI, ARC definite or probable stent thrombosis, urgent revascularization, MACEs	DM + AF + PCI	6 mo	Apixaban + P2Y12 inhibitor vs Warfarin + ASA + P2Y12 inhibitor
Vranckx 2019^[18]^	ISTH major bleeding, major bleeding, MACEs, fatal bleeding, intracranial bleeding	DM + AF + PCI	12 mo	Endoxaban + P2Y12 inhibitor vs Warfarin + ASA + P2Y12 inhibitor
Wang 2019^[19]^	MACCE, any bleeding, major bleeding	DM + AF + PCI	12 mo	New oral anticoagulant + P2Y12 inhibitor vs Warfarin + ASA + P2Y12 inhibitor

### 2.6 Data extraction and quality assessment

Data were extracted by 2 independent reviewers. Important information such as the adverse cardiovascular and bleeding outcomes which were reported in the original studies, the duration of follow-up, the number of DM participants who were assigned to the DT and TT groups respectively, the type of oral anticoagulants, the comorbidities which were reported, the year of publication, the type of study (randomized trial or observational cohort), the number of events in each outcome category were carefully extracted. Any disagreement was carefully discussed and resolved by consensus.

The methodological quality of the randomized trials was assessed by the recommendations suggested by the Cochrane collaboration^[13]^ whereas the methodological quality of the observational cohort was assessed by the Newcastle Ottawa Scale (NOS).^[14]^

### 2.7 Statistical analysis

This meta-analysis was carried out by the RevMan version 5.4 software. Heterogeneity was assessed by the Q statistic test. A *P* value less or equal to .05 was considered statistically significant. Heterogeneity was further assessed by the *I*^2^ statistic test. The smaller the *I*^2^ value, the lower the heterogeneity.

A fixed statistical effect model was used if *I*^2^ was below 50% whereas a random statistical effect model was used if the *I*^2^ value was above 50%.

Risk ratios (RR) with 95% confidence intervals (CI) were used to represent the data and interpret the analysis.

Sensitivity analysis was carried out using the exclusion method. One by one each study was eliminated and a new analysis was carried out each time to observe any significant change compared to the main results.

Since this analysis consisted of a small volume of studies, publication bias was visually assessed through funnel plots.

### 2.8 Compliance with ethical guidelines

This article is based on previously conducted studies and does not contain any studies with human participants or animals performed by any of the authors.

## 3 Results

### 3.1 Search outcomes

The Preferred Reporting Items in Systematic Reviews and Meta-Analyses (PRISMA) reporting guideline was followed.^[15]^ A total number of 2975 publications were obtained. However, based on their titles, an initial elimination was carried out and irrelevant titles were directly eliminated. Among the remaining publications, a second elimination was carried out after a careful assessment of the abstracts and titles of the remaining publications.

Finally, 208 full-text articles were assessed for eligibility. Based on the inclusion and exclusion criteria, further eliminations were carried out:

- Review articles (systematic reviews, literature reviews, pooled analyses and meta-analyses) (n = 9);
- Case studies (n = 21);
- Studies that did not compare DT (Non-vitamin K antagonist plus a P2Y12 inhibitor) vs TT (aspirin, a P2Y12 inhibitor and a vitamin K antagonist) (n = 23);
- Studies that were not based on PCI (n = 44);
- Studies that involved data which could not be used in this analysis (n = 6);
- Studies that did not report cardiovascular and bleeding outcomes (n = 12);
- Studies that involved the same trial, or were duplicates and were repeatedly found in different search databases (n = 88).

**Figure 1:**
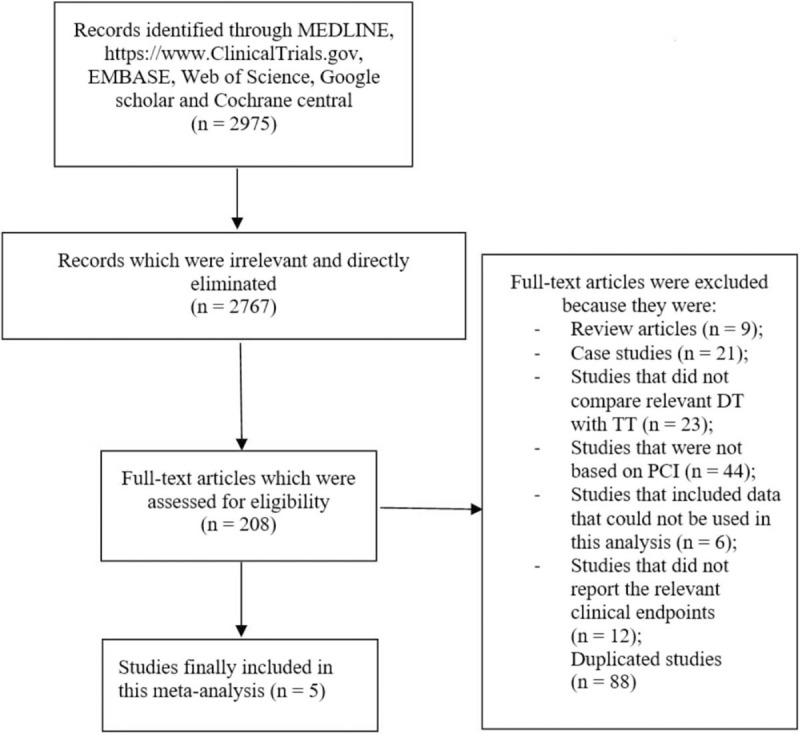
Flow diagram showing the study selection. From the searched databases, a total number of 2975 publications were obtained. However, based on their titles, and after carefully reviewing the titles and abstracts, an elimination was carried out and irrelevant publications were eliminated. Two hundred eight full-text articles were assessed for eligibility. Based on the inclusion and exclusion criteria, further eliminations were carried out as shown in Figure 1.

Finally, only a total number of 5 studies (4 randomized controlled trials and 1 observational cohort)^[12,16–19]^ were included in the final meta-analysis. The flow diagram showing the study selection was illustrated in Figure 1.

### 3.2 Main and baseline features of the studies and participants

A total number of 4970 participants were included in this analysis whereby 2456 participants were assigned to the DT group and 2514 participants were assigned to the TT group as shown in Table 2. The enrollment period varied from year 2006 to year 2018. Four studies were randomized trials, whereas 1 study was an observational cohort.

**Table 2:** Main features of the studies which were included in this analysis.

Studies	DM participants assigned to the DT group (n)	DM participants assigned to the TT group (n)	Type of study	Year of participants enrollment	Methodological assessment: Bias risk score
Cannon2017	622	674	RCT	2014–2016	B
Gibson2016	709	706	RCT	2013–2015	B
Lopes2019	842	836	RCT	2015–2018	B
Vranckx2019	259	258	RCT	2017–2018	B
Wang2019	24	40	OC	2006–2016	B
Total no of patients (n)	2456	2514			

Following a methodological assessment, the studies were allotted a bias risk grade as shown in Table 2.

The baseline features have been listed in Table 3. The mean age of the participants varied from 69.0 to 73.1 years. The percentage of male participants varied from 70.0% to 78.3%. The percentage of participants who were revascularized by drug eluting stents varied from 57.6% to 84.4%. The percentages of participants with comorbidities have also been listed in Table 3.

**Table 3:** Baseline features of the participants.

Studies	Age (years)	Males (%)	DL (%)	HBP (%)	DES (%)
	DT/TT	DT/TT	DT/TT	DT/TT	DT/TT
Cannon2017	70.1/70.3	75.9/77.1	–	–	81.8/84.4
Gibson2016	70.4/69.9	74.5/73.4	–	–	65.4/66.5
Lopes2019	70.4/70.9	70.9/71.1	–	88.6/88.0	–
Vranckx2019	69.0/70.0	74.0/75.0	66.0/64.0	90.0/91.0	–
Wang2019	72.7/73.1	78.3/70.0	46.4/55.0	66.7/76.3	57.6/60.3

## 4 Results of this analysis

**Figure 2:**
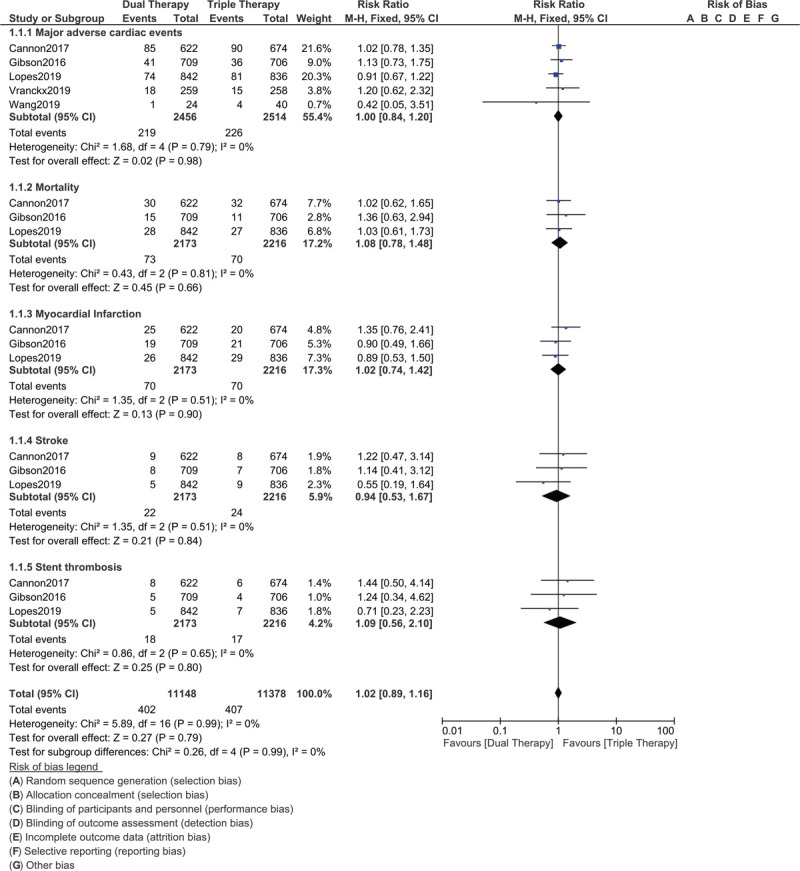
Efficacy of dual therapy with a non-vitamin K oral anticoagulant (NOAC) and a P2Y12 inhibitor vs triple therapy with aspirin, a P2Y12 inhibitor and a vitamin K antagonist for the treatment of atrial fibrillation patients with diabetes mellitus following percutaneous coronary intervention. Figure 2 shows that DT and TT were both equally effective in terms of adverse cardiovascular outcomes.

**Figure 3:**
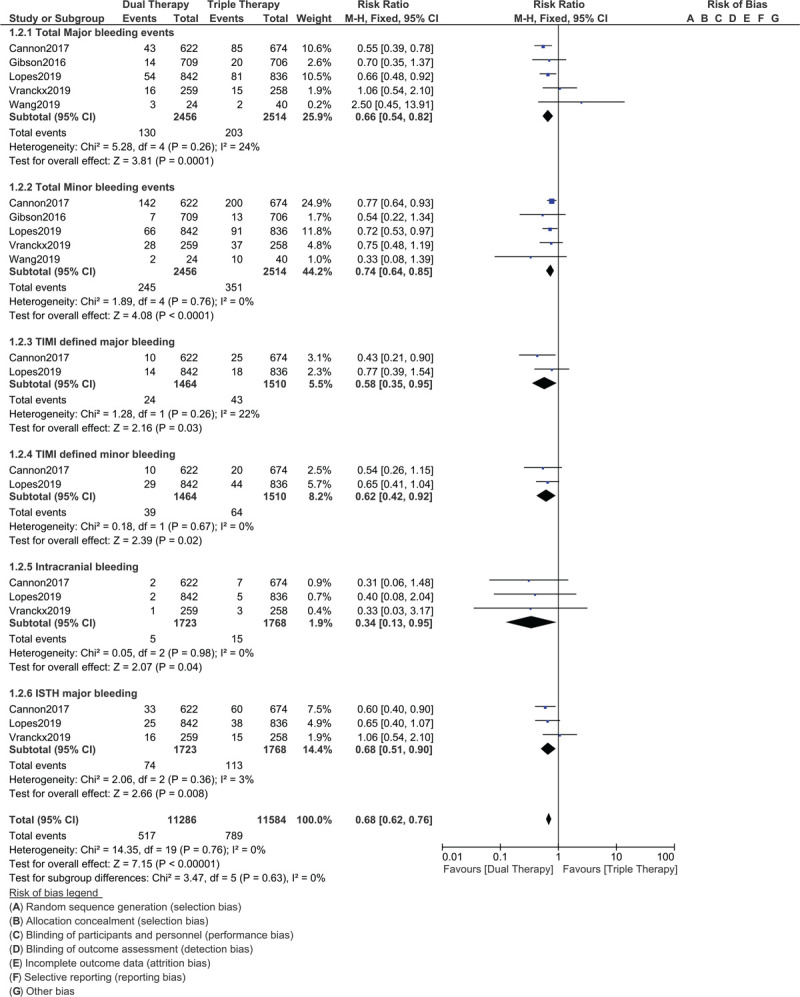
Safety of dual therapy with a NOAC and a P2Y12 inhibitor vs triple therapy with aspirin, a P2Y12 inhibitor and a vitamin K antagonist for the treatment of atrial fibrillation patients with diabetes mellitus (DM) following percutaneous coronary intervention. Figure 3 shows that DT was safer in comparison to TT in these patients.

Our current results showed that MACEs (RR: 1.00, 95% CI: 0.84–1.20; *P* = .98), mortality (RR: 1.08, 95% CI: 0.78–1.48; *P* = .66), MI (RR: 1.02, 95% CI: 0.74–1.42; *P* = .90), stroke (RR: 0.94, 95% CI: 0.53–1.67; *P* = .84) and stent thrombosis (RR: 1.09, 95% CI: 0.56–2.10; *P* = .80) were similar with DT vs TT in these DM patients with AF post PCI as shown in Figure 2. However, the risks for total major bleeding (RR: 0.66, 95% CI: 0.54–0.82; *P* = .0001), total minor bleeding (RR: 0.74, 95% CI: 0.64–0.85; *P* = .0001), TIMI defined major bleeding (RR: 0.58, 95% CI: 0.35–0.95; *P* = .03), TIMI defined minor bleeding (RR: 0.62, 95% CI: 0.42–0.92; *P* = .02), intra-cranial bleeding (RR: 0.34, 95% CI: 0.13–0.95; *P* = .04) and ISTH major bleeding (RR: 0.68, 95% CI: 0.51–0.90; *P* = .008) were significantly higher with TT as shown in Figure 3.

### 4.1 Sensitivity analysis and publication bias

Sensitivity analysis led to consistent results. Except that when study Cannon2017 was excluded, and a new analysis was carried out, intracranial bleeding (RR: 0.37, 95% CI: 0.10–1.40; *P* = .14) and ISTH bleeding (RR: 0.77, 95% CI: 0.52–1.15; *P* = .20) were not significantly different with DT vs TT.

The results have been summarized in Table 4.

**Table 4:** Results comparing dual vs triple therapy in DM patients with co-existing AF following PCI.

Outcomes	RR with 95% CI	*P* value	*I*^2^ value (%)
Major adverse cardiac events	1.00 [0.84–1.20]	.98	0
Mortality	1.08 [0.78–1.48]	.66	0
Myocardial infarction	1.02 [0.74–1.42]	.90	0
Stroke	0.94 [0.53–1.67]	.84	0
Stent thrombosis	1.09 [0.56–2.10]	.80	0
Total major bleeding events	0.66 [0.54–0.82]	.0001	24
Total minor bleeding events	0.74 [0.64–0.85]	.0001	0
TIMI defined major bleeding	0.58 [0.35–0.95]	.03	22
TIMI defined minor bleeding	0.62 [0.42–0.92]	.02	0
Intracranial bleeding	0.34 [0.13–0.95]	.04	0
ISTH major bleeding	0.68 [0.51–0.90]	.008	3

**Figure 4:**
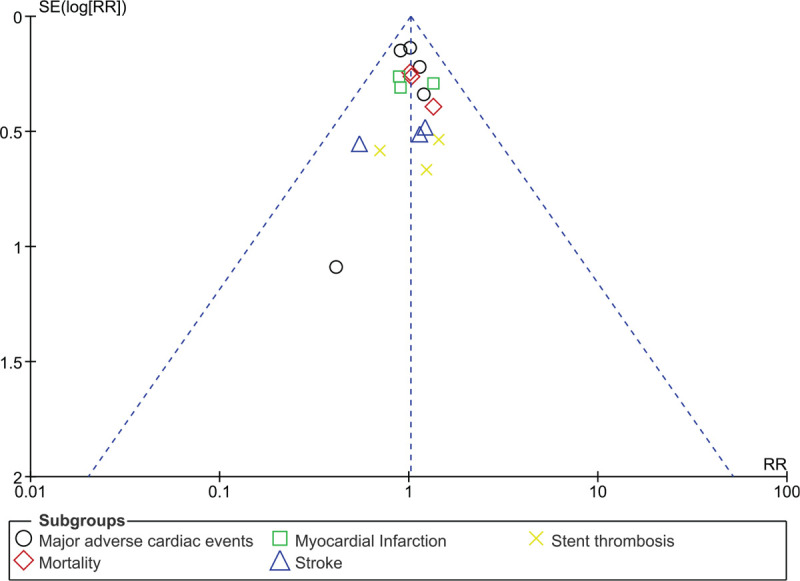
Funnel plot representing publication bias (A). The funnel plot A did not show any evidence of publication bias across the several studies that assessed the cardiovascular outcomes between DT and TT in these patients with atrial fibrillation and diabetes mellitus.

**Figure 5:**
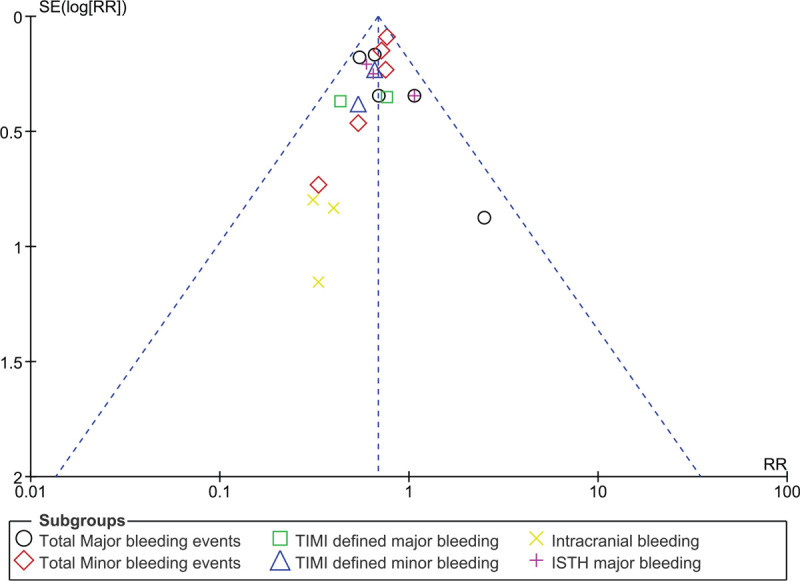
Funnel plot representing publication bias (B). The funnel plot B did not show any evidence of publication bias across the several studies that assessed bleeding/safety outcomes between DT and TT in these patients with atrial fibrillation and diabetes mellitus.

Figures 4 and 5 (funnel plots) showed a low evidence of publication bias across the studies which assessed the clinical outcomes between DT and TT in these DM patients with co-existing AF.

## 5 Discussion

Our current analysis showed that DT with an oral non-vitamin K antagonist plus a P2Y12 inhibitor was associated with significantly less major and minor bleedings including TIMI defined major and minor bleedings, ISTH major bleeding and intracranial bleeding, with similar cardiovascular outcomes compared to the TT (aspirin, P2Y12 inhibitor, and a vitamin K antagonist) in DM patients with co-existing AF following PCI. This current analysis showed that DT was safer and equally effective in these DM patients with AF.

Similarly, a recently published article showed DT not to increase the risk of MACEs but instead significantly reduced bleeding events compared to TT in AF patients who were revascularized by PCI.^[20]^ Our current analysis consisted of patients with DM. However, this newly published paper consisted of the general population of patients with CVD and involved over 10 000 participants.

The PIONEER study also supports the results of this current analysis.^[21]^ In their study, the authors showed that among patients with AF who were treated by PCI, administration of a non-vitamin K antagonist, more precisely rivaroxaban 15 mg once daily plus a P2Y12 inhibitor or rivaroxaban 2.5 mg twice daily plus DAPT was associated with reduced bleeding events compared to the standard of care vitamin K antagonist plus DAPT.

Other anti-thrombotic regimens have also been tried. The Cardiovascular Outcomes for People using Anticoagulation Strategies (COMPASS) trial compared DT with low dose rivaroxaban (2.5 mg) plus aspirin 100 mg versus aspirin 100 mg monotherapy following PCI.^[22]^ The authors demonstrated that DT significantly reduced MACEs and mortality, however, major bleeding was increased in comparison to aspirin monotherapy in these patients. Another study compared DT (rivaroxaban 2.5 mg plus aspirin 100 mg) versus rivaroxaban 5 mg monotherapy and aspirin 100 mg monotherapy respectively.^[23]^ It was found that the former decreased vascular events and mortality, but increased major bleeding without any increase in intracranial or fatal bleeding in comparison to rivaroxaban or aspirin monotherapy respectively. Nevertheless, our current analysis was different in regimen, comparing DT (non-vitamin K antagonist plus P2Y12 inhibitor) with TT (aspirin, P2Y12 inhibitor and vitamin K antagonist) for the treatment of DM patients with co-existing AF after PCI.

At last, we would like to point out that due to the development of aspirin and clopidogrel hypo-responsiveness especially in patients with DM, which might be due to platelet hyperactivity in this same category of patients, new combinations of DT should emerge in the coming years. Studies have shown that platelets have an increased response to procoagulants in patients with DM. In patients with DM, platelet hyperactivity, in the presence of oxidative stress, have been found to increase the progression of thrombotic and cardiovascular events.^[24]^ Therefore, a combination of DT with a NOAC and a P2Y12 inhibitor for the treatment of DM patients with AF postPCI might be included in future guidelines.

## 6 Limitations

This study has limitations. First of all, due to the limited number of participants with DM, the analysis might not provide robust results. Secondly, 2 studies had different follow up time periods (6 and 14 months respectively) compared to the other studies which had a follow up time period of 12 months. Moreover, in 1 study (Gibson2016), the number of patients with DM was not known and therefore, the general participants were used. Also, it should be noted that due to the fact that almost no study specifically involving participants with DM has been published on this aspect till date, we had to extract DM participants from original studies which included patients from the general population with ACS who were treated with DT versus TT. Another limitation was the fact that the NOAC was different in each study. One study involved dabigatran, another study involved rivaroxaban, 1 study included patients who were treated with apixaban, and another study used endoxaban. Also, the dosage of the new oral anticoagulants was not taken into consideration and this could have had an impact on the outcomes.

## 7 Conclusions

DT with a NOAC and a P2Y12 inhibitor was associated with significantly less bleeding events without increasing the adverse cardiovascular outcomes when compared to TT with aspirin, a P2Y12 inhibitor and a Vitamin K antagonist for the treatment of DM patients with co-existing AF following PCI. Hence, DT is comparable in efficacy, but safer compared to TT. However, due to the limitations of this study, including the small number of participants, this interesting hypothesis will have to be confirmed in future studies.

## Acknowledgments

All named authors meet the International Committee of Medical Journal Editors (ICMJE) criteria for authorship for this article, take responsibility for the integrity of the work as a whole, and have given their approval for this version to be published.

## Author contributions

The authors QW and KY were responsible for the conception and design, acquisition of data, analysis and interpretation of data, drafting the initial manuscript and revising it critically for important intellectual content. Authors QW wrote this manuscript. All the authors agreed to and approved the manuscript as it is.

**Conceptualization:** Qiang Wang, Keping Yang.

**Data curation:** Qiang Wang, Keping Yang.

**Formal analysis:** Qiang Wang, Keping Yang.

**Funding acquisition:** Qiang Wang, Keping Yang.

**Investigation:** Qiang Wang, Keping Yang.

**Methodology:** Qiang Wang, Keping Yang.

**Project administration:** Qiang Wang, Keping Yang.

**Resources:** Qiang Wang, Keping Yang.

**Software:** Qiang Wang, Keping Yang.

**Supervision:** Qiang Wang, Keping Yang.

**Validation:** Qiang Wang, Keping Yang.

**Visualization:** Qiang Wang, Keping Yang.

**Writing – original draft:** Qiang Wang.

**Writing – review & editing:** Qiang Wang.
